# Common Hepatic Branch of Vagus Nerve-Dependent Expression of Immediate Early Genes in the Mouse Brain by Intraportal L-Arginine: Comparison with Cholecystokinin-8

**DOI:** 10.3389/fnins.2017.00366

**Published:** 2017-06-28

**Authors:** Daisuke Yamada, Peter Koppensteiner, Saori Odagiri, Megumi Eguchi, Shun Yamaguchi, Tetsuya Yamada, Hideki Katagiri, Keiji Wada, Masayuki Sekiguchi

**Affiliations:** ^1^Department of Degenerative Neurological Diseases, National Center of Neurology and Psychiatry, National Institute of NeuroscienceTokyo, Japan; ^2^Department of Morphological Neuroscience, Graduate School of Medicine, Gifu UniversityGifu, Japan; ^3^Center for Highly Advanced Integration of Nano and Life Sciences, Gifu UniversityGifu, Japan; ^4^Precursory Research for Embryonic Science and Technology (PRESTO), Japan Science and Technology AgencySaitama, Japan; ^5^Department of Metabolism and Diabetes, Graduate School of Medicine, Tohoku UniversityMiyagi, Japan; ^6^CREST, Japan Agency for Medical Research and DevelopmentTokyo, Japan

**Keywords:** vagus nerve, hepatic branch, L-arginine, cholecystokinin-8, insular cortex, hypothalamus

## Abstract

Information from the peripheral organs is thought to be transmitted to the brain by humoral factors and neurons such as afferent vagal or spinal nerves. The common hepatic branch of the vagus (CHBV) is one of the main vagus nerve branches, and consists of heterogeneous neuronal fibers that innervate multiple peripheral organs such as the bile duct, portal vein, paraganglia, and gastroduodenal tract. Although, previous studies suggested that the CHBV has a pivotal role in transmitting information on the status of the liver to the brain, the details of its central projections remain unknown. The purpose of the present study was to investigate the brain regions activated by the CHBV. For this purpose, we injected L-arginine or anorexia-associated peptide cholecystokinin-8 (CCK), which are known to increase CHBV electrical activity, into the portal vein of transgenic *Arc*-*dVenus* mice expressing the fluorescent protein Venus under control of the activity-regulated cytoskeleton-associated protein (Arc) promotor. The brain slices were prepared from these mice and the number of Venus positive cells in the slices was counted. After that, c-Fos expression in these slices was analyzed by immunohistochemistry using the avidin-biotin-peroxidase complex method. Intraportal administration of L-arginine increased the number of Venus positive or c-Fos positive cells in the insular cortex. This action of L-arginine was not observed in CHBV-vagotomized *Arc-dVenus* mice. In contrast, intraportal administration of CCK did not increase the number of c-Fos positive or Venus positive cells in the insular cortex. Intraportal CCK induced c-Fos expression in the dorsomedial hypothalamus, while intraportal L-arginine did not. This action of CCK was abolished by CHBV vagotomy. Intraportal L-arginine reduced, while intraportal CCK increased, the number of c-Fos positive cells in the nucleus tractus solitarii in a CHBV-dependent manner. The present results suggest that the CHBV can activate different brain regions depending on the nature of the peripheral stimulus.

## Introduction

Information on peripheral organ status is thought to be transmitted to the brain without awareness, and this process, designated “interoception,” is important for maintenance of homeostasis in animals, including humans (Craig, 2002; Damasio and Carvalho, 2013). Chemical and electrical signals both act in this process, with the former involving humoral factors released into body fluids from the peripheral organs. The latter occurs through the control of spike firing in afferent peripheral neurons and subsequent transmitter release to central neurons from the nerve endings of peripheral neurons (Craig, 2002; Damasio and Carvalho, 2013). Two distinct afferent nervous systems, cranial (primarily vagus) and spinal nerves, serve as the afferent peripheral neurons for interoception (Craig, 2002; Saper, 2002; Damasio and Carvalho, 2013). The primary somatosensory cortex is the region in which visceral sensory information received *via* the spinal cord is represented. Meanwhile, vagal information mainly enters the brain at the nucleus tractus solitarii (NTS), and NTS neurons deliver vagal neuronal signals to various central regions using direct efferent connections or indirect neuronal circuits (Cechetto and Saper, 1987).

The common hepatic branch of the vagus (CHBV) is one of the main vagus nerve branches. It diverges from the left subdiaphragmatic vagus trunk immediately caudal to the diaphragm (Berthoud and Neuhuber, 2000; Puizillout, 2005). There are approximately 3,000 nerve fibers in the rat CHBV, of which 2,200 are afferent fibers and only 200 are efferent fibers. The remaining 600 are non-vagal adventitial fibers such as sympathetic post-ganglion or dorsal root afferent fibers (Prechtl and Powley, 1990). At the periphery, the CHBV projects to the bile duct, portal vein, paraganglia, and gastroduodenal tract. Consequently, the branch consists of heterogeneous nerve fibers with respect to the projections of their peripheral axons. In rats, vagal afferent terminals are not found in the hepatic parenchyma (Berthoud et al., 1992; Berthoud and Neuhuber, 2000). It is inferred that the CHBV plays pivotal roles in interoception based on its high content of afferent fibers. Indeed, it is known that changes in the condition of the liver are transmitted to the brain *via* the CHBV and stimulate homeostatic metabolic control in the mouse (Uno et al., 2006, 2015; Imai et al., 2008; Tsukita et al., 2012).

The central nervous system (CNS) axons of neurons in the CHBV are known to project to the NTS (Rogers and Hermann, 1983). The predominant projection sites are the left subnucleus gelatinosus, medial division of the left solitary nucleus, and left lateral edge of the area postrema (Rogers and Hermann, 1983). When a signal arrives at the NTS *via* vagal afferents, it is transmitted to other brain regions through neural connections. Many brain regions, such as the dorsal motor nucleus of the vagus, parabrachial nucleus, hypothalamus, amygdala, bed nucleus of the stria terminalis, and insular cortex, are known to receive direct neuronal fibers from the NTS (Puizillout, 2005). However, it remains unknown whether the heterogeneity of the fibers in the CHBV is reflected in the activation of distinct brain regions.

Previous studies have shown the presence of chemical substances that facilitate electrical activity of the CHBV when administered into the hepatic portal vein. These factors include insulin (Niijima, 1984), glucagon-like peptide-1 (Nishizawa et al., 1996), interleukin-1β (Niijima, 1996), somatostatin (Nakabayashi, 1997), linoleic acid (Randich et al., 2001), L-arginine, and seven other amino acids (L-arginine being the most potent; Niijima and Meguid, 1995). While cholecystokinin-8 (CCK) injection into the portal vein also increases CHBV activity (Horn and Friedman, 2004), this action of CCK is not observed in all CHBV filaments, and generally detected in about half of filaments tested (Cox and Randich, 1997).

The aim of the present study was to clarify whether the heterogeneity of the fibers in the CHBV results in activation of distinct brain regions. This knowledge would be helpful toward understanding the role of the CHBV in interoception. For this purpose, we compared the effects of two different CHBV stimulants, L-arginine and CCK, on the expression of two neuronal activity-dependent immediate early genes (IEGs) in the brain. It has been shown that peripheral CCK plays a physiological role in the regulation of food intake (Gibbs et al., 1973), and that the amino acids that exhibit excitatory or inhibitory action on the activity of afferent fibers of CHBV may influence appetite (Niijima and Meguid, 1995). Specifically, L-arginine and CCK were injected into the portal vein of *Arc-dVenus* transgenic mice (Eguchi and Yamaguchi, 2009), in which expression of the fluorescent protein Venus is induced by activation of the activity-regulated cytoskeleton-associated protein (Arc) promoter (Lyford et al., 1995). *Arc* is an IEG expressed in response to neural activity, and its expression in the hippocampus is strongly induced by high-frequency stimulation that elicits long-term potentiation of the synaptic response in perforant path synapses (Lyford et al., 1995). We prepared brain slices from L-arginine- or CCK-injected *Arc-dVenus* transgenic mice to perform fluorescent observation for Venus, and these brain slices were subsequently examined the expression of another IEG, c-Fos (Hughes and Dragunow, 1995) by immunohistochemistry using the avidin-biotin-peroxidase complex method. We particularly focused on the expression of these IEGs in the insular cortex, because this brain region is known as the “visceral cortex” and receives neural signals from the viscera originating in the vagus nerves (Cechetto and Saper, 1987). The dorsomedial hypothalamus, one of the regions showing increased c-Fos expression after intraperitoneal administration of CCK (Kobelt et al., 2006), was also analyzed for c-Fos expression induced by L-arginine or CCK.

## Materials and methods

### Animals

Total of 64 *Arc-dVenus* mice were used. All *Arc-dVenus* mice were 7–10 weeks of age at the time of the experiments. The mice were housed at 4–5 animals per cage under controlled temperature (25 ± 1°C) and lighting (12-h/12-h light/dark cycle), and provided with food and water *ad libitum*. All experimental procedures were conducted in strict accordance with the regulations of the National Institute of Neuroscience (Tokyo, Japan) for animal experiments, and were approved by the Institutional Animal Investigation Committee. All efforts were made to minimize animal suffering and to reduce the number of animals used. *Arc-dVenus* mice were generated as previously described (Eguchi and Yamaguchi, 2009). The mice were male transgenic heterozygotes, bred and housed at the Animal Center of the National Institute of Neuroscience, National Center of Neurology and Psychiatry (Tokyo, Japan).

### Drugs

L-Arginine hydrochloride (Arg), L-*N*^G^-nitroarginine methyl ester (L-NAME), L-phenylalanine (Phe), and CCK were purchased from Sigma-Aldrich (St. Louis, MO), and dissolved in saline. Arg was used to avoid altering the pH of the solution. Arg, L-NAME, and Phe were dissolved in saline at 150 mM. CCK was dissolved in saline at 0.44 mM. For dose-response experiments, 10 mM Arg and 2.20 mM CCK solutions were also used.

### Anesthesia

Special attention was paid to minimizing Venus and c-Fos expression artifacts. In particular, as c-Fos and Arc expression are stimulated by exposure to novel environments (Ramirez-Amaya et al., 2005), we devised a method in which the mice were anesthetized in their home cage. Each mouse was transferred from their home cage to a quasi-home cage at 24 h before the experiments. The quasi-home cage was constructed from the same plastic, was the same shape as the home cage, and was filled with wood chips at 1-cm depth, consistent with the home cage. A 4-mm diameter hole was made near the bottom of one wall, and at the time of anesthesia, the outlet of a sevoflurane anesthesia apparatus was connected to the hole. The mice were thus anesthetized in a familiar environment without necessity for transfer to a novel environment. The mice were fasted the night before to avoid the possible variation in the IEG expression by feeding behavior.

### Portal vein injection

Once anesthetized, the mouse was picked up and its head was inserted into a tube through which sevoflurane was continuously administered. Under stable anesthesia, a laparotomy was carried out in the dorsal position and the hepatic portal vein was exposed. Saline, Arg, Arg+L-NAME, Phe, or CCK was injected into the portal vein over 30 s using a 30-gauge needle with a 100-μL Hamilton-syringe. The injected dose of Arg and CCK was 0.61 mmol/kg and 1.75 nmol/kg, respectively, based on previous studies showing enhanced CHBV electrical activity after intraportal administration of these compounds (Niijima and Meguid, 1995; Cox and Randich, 1997; Horn and Friedman, 2004). The injected dose of Phe was 0.61 mmol/kg. In the dose-response experiments, 0.04 mmol/kg Arg and 9.41 nmol/kg CCK were used. After injection, the needle was carefully removed to avoid induction of bleeding, and the abdominal muscles and skin were separately sutured. Any mouse with significant bleeding from the portal vein was removed from the experiment. Following the procedure, each mouse was transferred back to its home cage, and allowed to recover naturally from the anesthesia over several minutes.

### Slice preparation and immunohistochemistry

At 90 and 180 min after surgery, to allow for expression of Venus (180 min, Eguchi and Yamaguchi, 2009) and c-Fos (90 and 180 min, Young et al., 1991; Hughes and Dragunow, 1995), each mouse was deeply anesthetized with fluothane gas, and transcardially perfused with phosphate-buffered saline (PBS) and 4% paraformaldehyde. The brain was removed from the skull, and post-fixed overnight in 4% paraformaldehyde. Coronal slices (50-μm thickness) were prepared using a vibratome (Sekiguchi et al., 2009). Then, slices were selected according to their bregma level (Paxinos and Franklin, 2001) and were put on the cover slips without sealing and Venus expression was observed directly using a BZ-X710 All-in-One Fluorescence Microscope (Keyence, Tokyo, Japan). A digital file containing a brain atlas (Paxinos and Franklin, 2001) was superimposed over each photomicrograph to specify the brain regions. The number of Venus positive cells was counted manually by an observer blinded to the treatment conditions. The counts from an individual animal (only one slice for each bregma level) are presented for a single data point.

Slices to be used in c-Fos immunostaining were selected from the slices used in analysis of Venus expression, according to their bregma level (Paxinos and Franklin, 2001). These slices were incubated in PBS containing 3% normal goat serum (Vector Laboratories, Burlingame, CA) and 0.3% Triton X-100 for 1 h at room temperature, treated with anti-c-Fos antibodies (1:5,000 dilution; Santa Cruz Biotechnology, Dallas, TX) overnight at 4°C in PBS, and washed with PBS. The signals were visualized using the avidin-biotin-peroxidase complex method (Vector Laboratories, Burlingame, CA) as previously described (Odagiri et al., 2012). The number of c-Fos positive cells was counted manually by an observer blinded to the treatment conditions. The counts from an individual animal (only one slice for each bregma level) are presented for a single data point.

### CHBV vagotomy

Surgery for selective CHBV vagotomy was carried out as previously described (Uno et al., 2006, 2015). Briefly, a laparotomy was performed in the dorsal position as described above. The descending ventral esophagus was revealed by gently retracting the stomach after severing the gastrohepatic ligament. The CHBV, diverging from the ventral subdiaphragmatic vagal trunk immediately caudal to the diaphragm, was then transected near the vagal trunk. The abdominal muscle and skin were separately sutured. The mice were used for portal vein injection at 7 days after surgery. The hepatic branch of the splanchnic nerve was left intact (Uno et al., 2015).

### Statistics

Data were expressed as mean ± standard error of mean (SEM) and analyzed by one-way analysis of variance (ANOVA). Significant ANOVA results were analyzed by a *post hoc* Bonferroni test. *P* < 0.05 was considered statistically significant.

## Results

### Intraportal Arg, but not CCK, increases venus positive cells in the insular cortex in a CHBV-dependent manner

**Figure 1:**
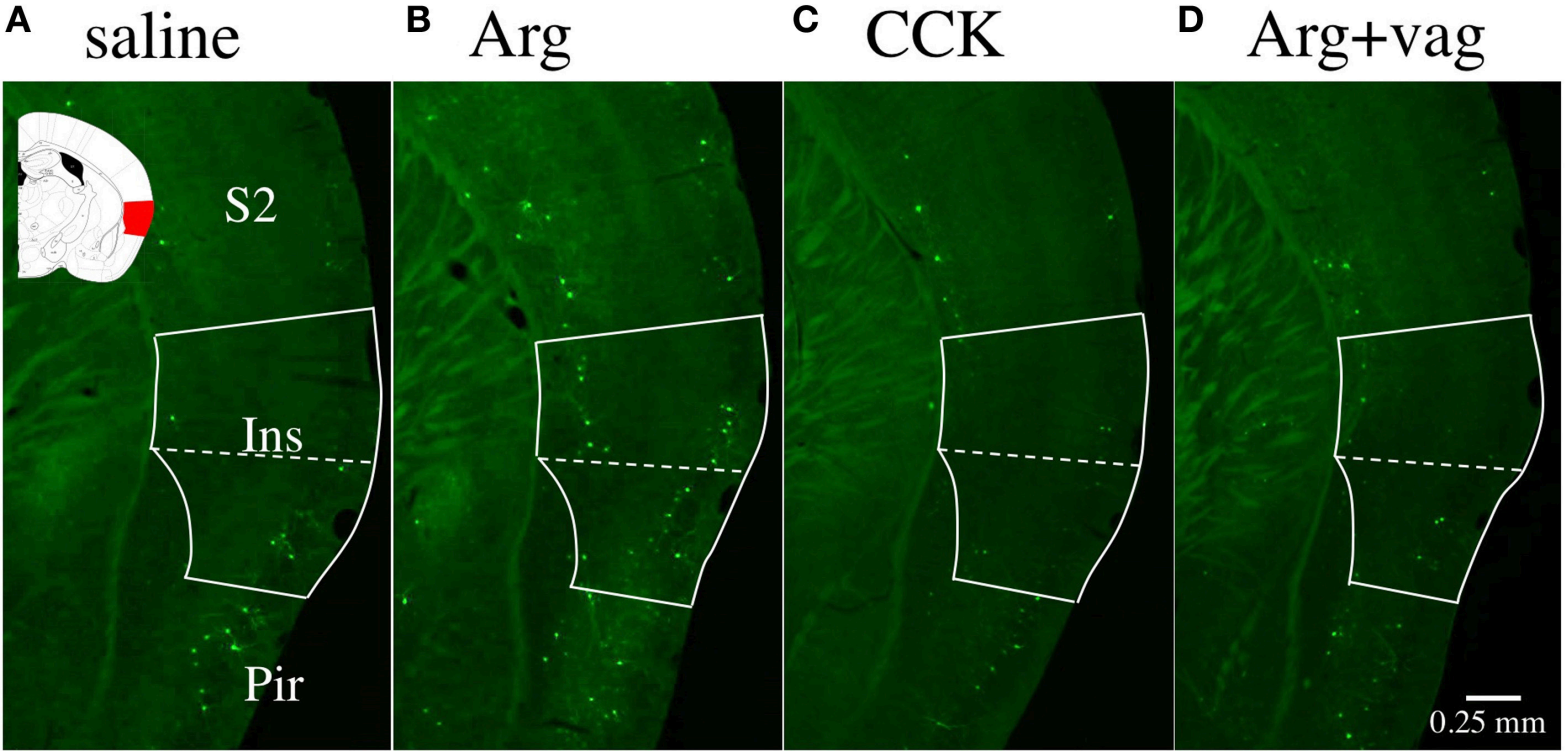
Fluorescence photomicrographs of brain coronal slices prepared from *Arc-dVenus* mice intraportally injected with saline **(A)**, Arg (**B**, 0.61 mmol/kg), and CCK (**C**, 1.75 nmol/kg), and a vagotomized *Arc-dVenus* mouse similarly injected with Arg (**D**, 0.61 mmol/kg). The solid white lines indicate the area of the insular cortex, and the dotted lines indicate the border of the granular/dysgranular and agranular insular cortices. In the inset shown in **(A)**, the area of the insular cortex at bregma −1.06 mm is marked in red (Paxinos and Franklin, 2001). Note that Venus positive cells (bright green dots) were increased in the insular cortex of the Arg-injected non-vagotomized *Arc-dVenus* mouse. Ins, insular cortex; S2, secondary somatosensory cortex; Pir, piriform cortex.

Figure 1A shows a photomicrograph of part of a brain slice (bregma −1.06 mm) prepared from an *Arc-dVenus* mouse intraportally injected with saline. The inset is the corresponding area from the brain atlas of Paxinos and Franklin (2001). The solid white line indicates the area of the insular cortex, and the bright green dots are cells expressing the fluorescent protein Venus. Figures 1B,C show photomicrographs of brain slices from mice injected with Arg (0.61 mmol/kg) and CCK (1.75 nmol/kg), respectively. More Venus positive cells were observed in the insular cortex of the Arg-injected mouse (Figure 1B) than in the insular cortex of the saline-injected mouse (Figure 1A). No such increase was observed in the CCK-injected mouse (Figure 1C). Figure 1D shows a similar photomicrograph of a brain slice from an Arg (0.61 mmol/kg)-injected *Arc-dVenus* mouse that had undergone CHBV vagotomy 7 days earlier (Arg+vag). The number of Venus positive cells in the vagotomized mouse was similar to that in the saline-injected mouse (Figure 1A), but smaller than that in the Arg-injected non-vagotomized mouse (Figure 1B).

### Anterior-posterior distribution of venus positive cells in the insular cortex

**Figure 2:**
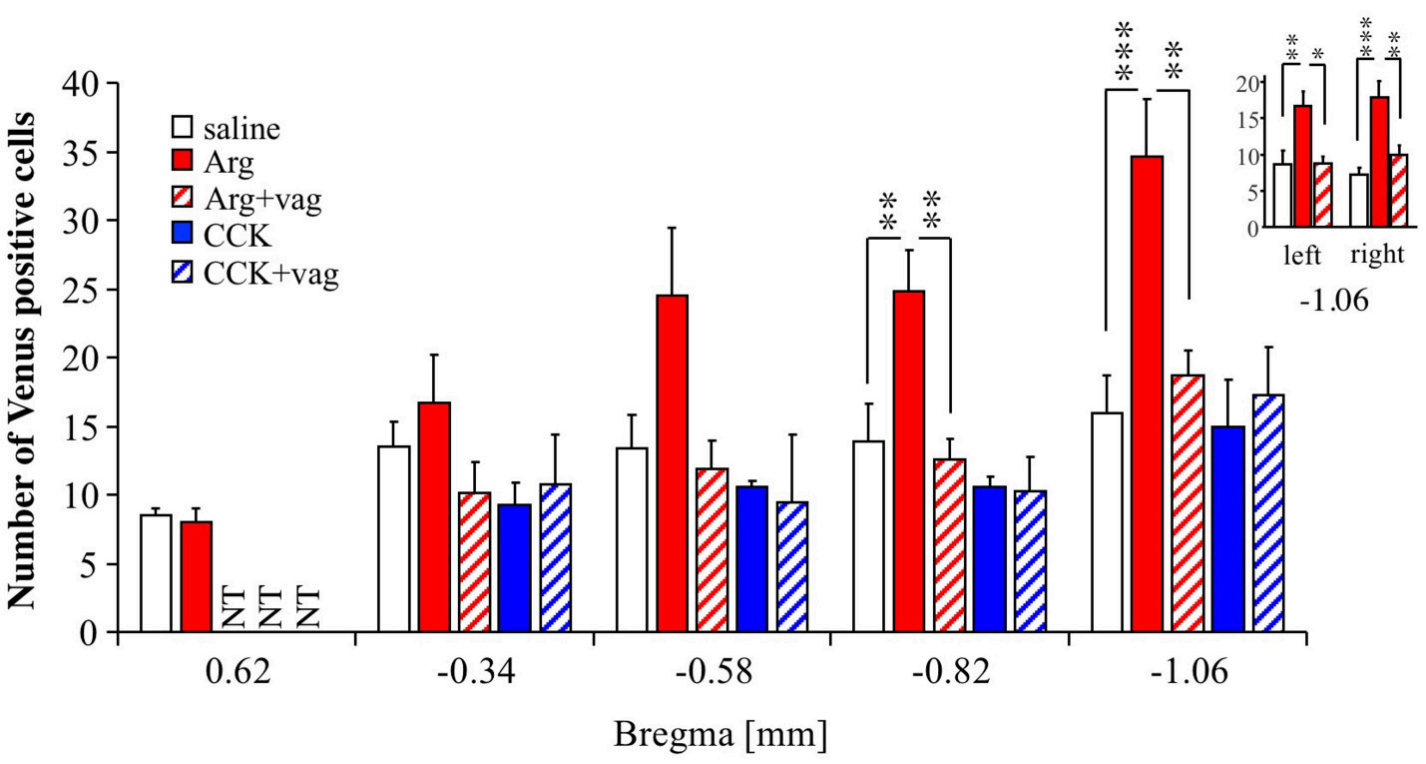
Anterior-posterior distribution of Venus positive cells in the insular cortex of *Arc-dVenus* mice, including bregma −1.06 mm. saline, Arg, and CCK refer to *Arc-dVenus* mice intraportally injected with saline (9 slices from 9 mice), Arg (0.61 mmol/kg, 8 slices from 8 mice), and CCK (1.75 nmol/kg, 4 slices from 4 mice), respectively. In addition, vagotomized (+vag) *Arc-dVenus* mice were similarly injected with the same doses of Arg (7 slices from 7 mice) and CCK (4 slices from 4 mice). Brain slices from the levels of bregma +0.62, −0.34, −0.58, −0.82, and −1.06 mm were prepared, and the numbers of Venus positive cells were counted. (inset) In the same slices (at bregma −1.06 mm) used in this experiment, the number of Venus positive cells was counted in left and right hemispheres separately. Data are shown as mean ± SEM. NT: not tested. ^*^P < 0.05, ^**^P < 0.01, ^***^P < 0.001 (*post hoc* Bonferroni test). Note that a highly significant increase in the number of Arg-induced Venus positive cells was observed in the posterior portions of the insular cortex, and abolished by CHBV vagotomy.

A previous study showed that the insular cortex in rats can be roughly divided into two areas, an anterior (taste) area and a posterior (visceral) area (Cechetto and Saper, 1987). In the case of mice, it can be roughly estimated that the insular cortex starts near bregma +2.4 mm and ends near bregma −1.2 mm, with the joining of the anterior commissure at approximately bregma +0.14 mm (Paxinos and Franklin, 2001). Taking this into account, we analyzed the effect of Arg (0.61 mmol/kg) on Venus expression at bregma +0.62, −0.34, −0.58, and −0.82 mm as well as at bregma −1.06 mm (Figure 2). The number of animals used in each treatment group was 9 for saline, 8 for Arg, 7 for Arg+vag, 4 for CCK, and 4 for CCK+vag. Bregma +0.62 mm is expected to correspond to the taste area (Cechetto and Saper, 1987). As shown in Figure 2, a significant Arg-induced increase in the Venus positive cell number was observed in the posterior portion of the insular cortex at bregma −0.82 mm (*P* < 0.01), as well as at bregma −1.06 mm (*P* < 0.001) [*F*_(4, 27)_ = 5.73, *P* = 0.0018 for bregma −0.82 mm; *F*_(4, 27)_ = 6.89, *P* = 0.0006 for bregma –1.06 mm; one-way ANOVA]. No significant increase was observed in the more anterior portions, such as bregma +0.62, −0.34, and −0.58 mm. The effect of Arg at bregma −0.82 and −1.06 mm was abolished in vagotomized mice (Figure 2). The action of Arg at bregma +0.62 mm in vagotomized mice was not tested (Figure 2, NT), because Arg did not induce a significant increase in the number of Venus positive cells in non-vagotomized mice. In contrast, intraportal CCK (1.75 nmol/kg) did not increase the number of Venus positive cells at bregma −0.34, −0.58, −0.82, and −1.06 mm. The action of CCK at bregma +0.62 mm was not tested (Figure 2, NT), because Arg did not induce a significant increase in the number of Venus positive cells in non-vagotomized mice. We also examined laterality of the CHBV-dependent action of intraportal Arg in the insular cortex. In the same slices (at bregma −1.06 mm) used in this experiment, the number of Venus positive cells were counted in left and right hemispheres separately. Similar action of intraportal Arg on Venus expression was observed both in left and right hemispheres (Figure 2-inset).

The above results suggest that the number of Venus positive cells is enhanced in the posterior insular cortex in response to intraportal Arg in a CHBV-dependent manner, which is consistent with the view that the posterior insular cortex contains a visceral area.

### Intraportal Arg, but not CCK, increases c-Fos positive cells in the insular cortex in a CHBV-dependent manner

**Figure 3:**
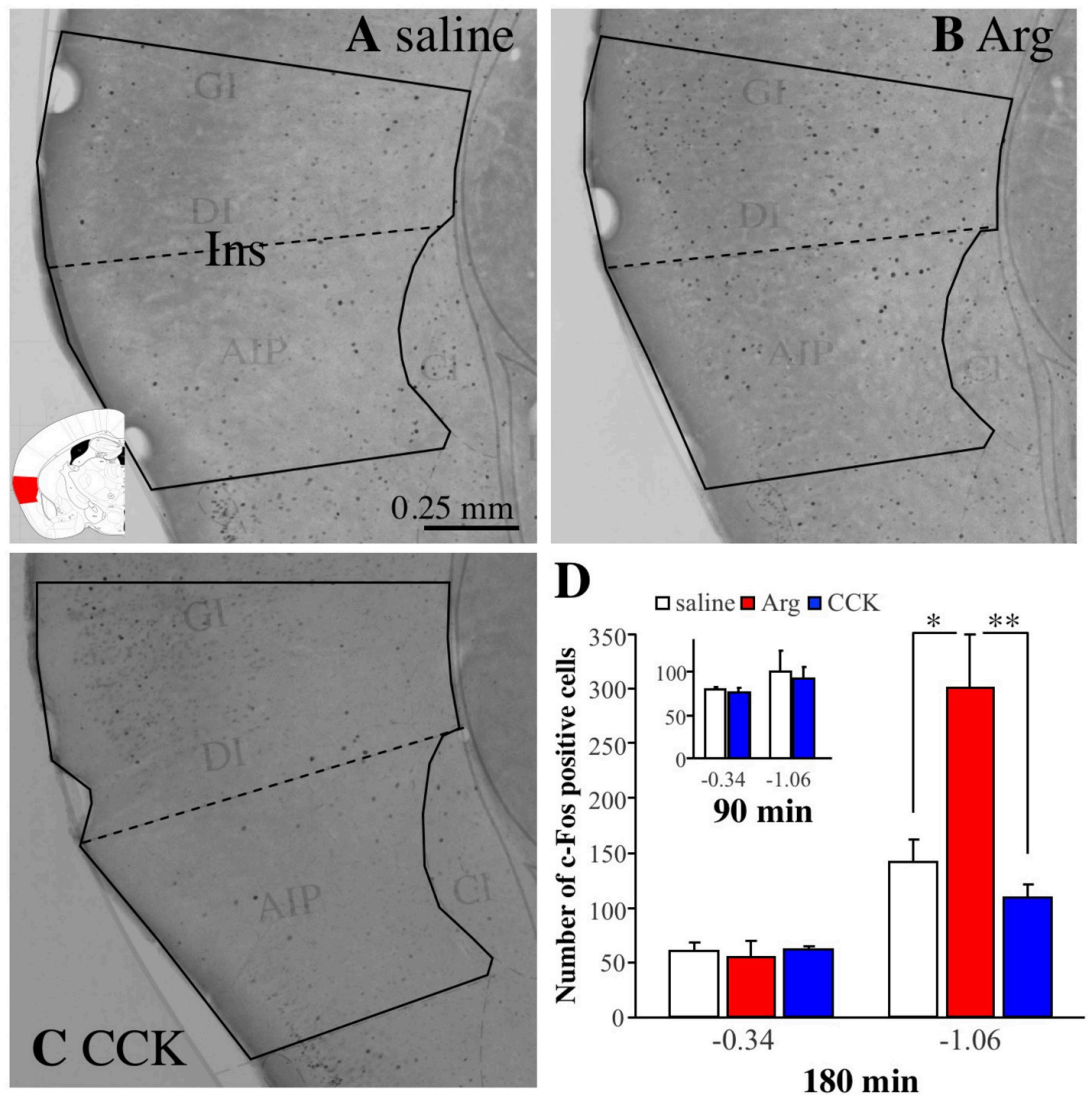
Immunostaining with anti-c-Fos antibodies of the insular cortex in brain coronal slices from *Arc-dVenus* mice (the slices used in Figure 2). The mice were intraportally injected with saline **(A)**, Arg (**B**, 0.61 mmol/kg), and CCK (**C**, 1.75 nmol/kg), and fixed at 180 min after the portal vein injection. The solid black lines indicate the area of the insular cortex, and the dotted lines indicate the border of the granular/dysgranular and agranular insular cortices. The brain slices were treated with anti-c-Fos antibodies and the signals were visualized using the avidin-biotin-peroxidase complex method. Note that c-Fos positive cells (black dots) were increased in the insular cortex of the Arg-injected mouse. Ins: insular cortex. **(D)** The number of c-Fos positive cells in mice intraportally injected with saline (5 slices from 5 mice), Arg (**B**, 0.61 mmol/kg, 5 slices from 5 mice), and CCK (**C**, 1.75 nmol/kg, 4 slices from 4 mice) at bregma levels of −0.34 and −1.06 mm. (**D** inset) The data from saline-injected (3 slices from 3 mice) and CCK-injected (1.75 nmol/kg, 5 slices from 5 mice) mice. These mice were fixed at 90 min after portal vein injection. Data are shown as mean ± SEM. ^*^P < 0.05, ^**^P < 0.01 (*post hoc* Bonferroni test).

Figure 3A shows a photomicrograph of part of a brain slice (bregma −1.06 mm) prepared from a mouse intraportally injected with saline. The solid black line indicates the area of the insular cortex, and the black dots are immunoreactive signals for c-Fos. Figures 3B,C show photomicrographs of brain slices from mice injected with Arg (0.61 mmol/kg) and CCK (1.75 nmol/kg), respectively. More c-Fos positive cells were observed in the insular cortex of the Arg-injected mouse (Figure 3B) than in the insular cortex of the saline-injected mouse (Figure 3A). No such increase was observed in the CCK-injected mouse (Figure 3C). Similar experiments were performed in 5 saline-injected, 5 Arg-injected, and 4 CCK-injected mice, and the mean values (+ SEM) obtained in each group are shown in Figure 3D. One-way ANOVA revealed significant variation in the data [*F*_(2, 11)_ = 9.06, *P* = 0.005]. A *post hoc* Bonferroni test revealed a significant difference between saline-injected mice and Arg-injected mice (*P* < 0.05). Intraportal CCK had no significant effect on the number of c-Fos positive cells (−1.06 mm in Figure 3D). In addition, we performed similar analysis at the bregma level of −0.34 mm, a more rostral position, because intraportal Arg does not increase the number of Venus positive cells at this bregma level. The number of c-fos positive cells was not increased either at this level (−0.34 mm in Figure 3D).

As we mentioned earlier, we chose a time point of 180 min after intraportal administration for the analysis of c-Fos expression because we wanted to analyze the expression of c-Fos in the same slice that was analyzed the expression of Venus (analyzed at 180 min post-administration). However, a number of studies have suggested that peak responses of c-Fos protein expression occur within 60–120 min post-stimuli (Hughes and Dragunow, 1995). Accordingly, we also tested a time point of 90 min after CCK administration (Figure 3D-inset) to check the possibility that the c-Fos levels related to CCK would likely have been on the decline or gone at 180 min after CCK administration. In this case, it is expected that the increase in expression of c-Fos can be observed at 90 min after CCK administration. The number of animals used was 3 for saline and 5 for CCK. As Figure 3D-inset shows, however, there was no increases in the c-Fos level at 90 min after CCK administration. These results suggest that intraportal CCK does not increase the number of c-Fos positive cells in the insular cortex at bregma −0.34 and −1.06 mm.

### Effects of Arg and CCK in other cortices

**Figure 4:**
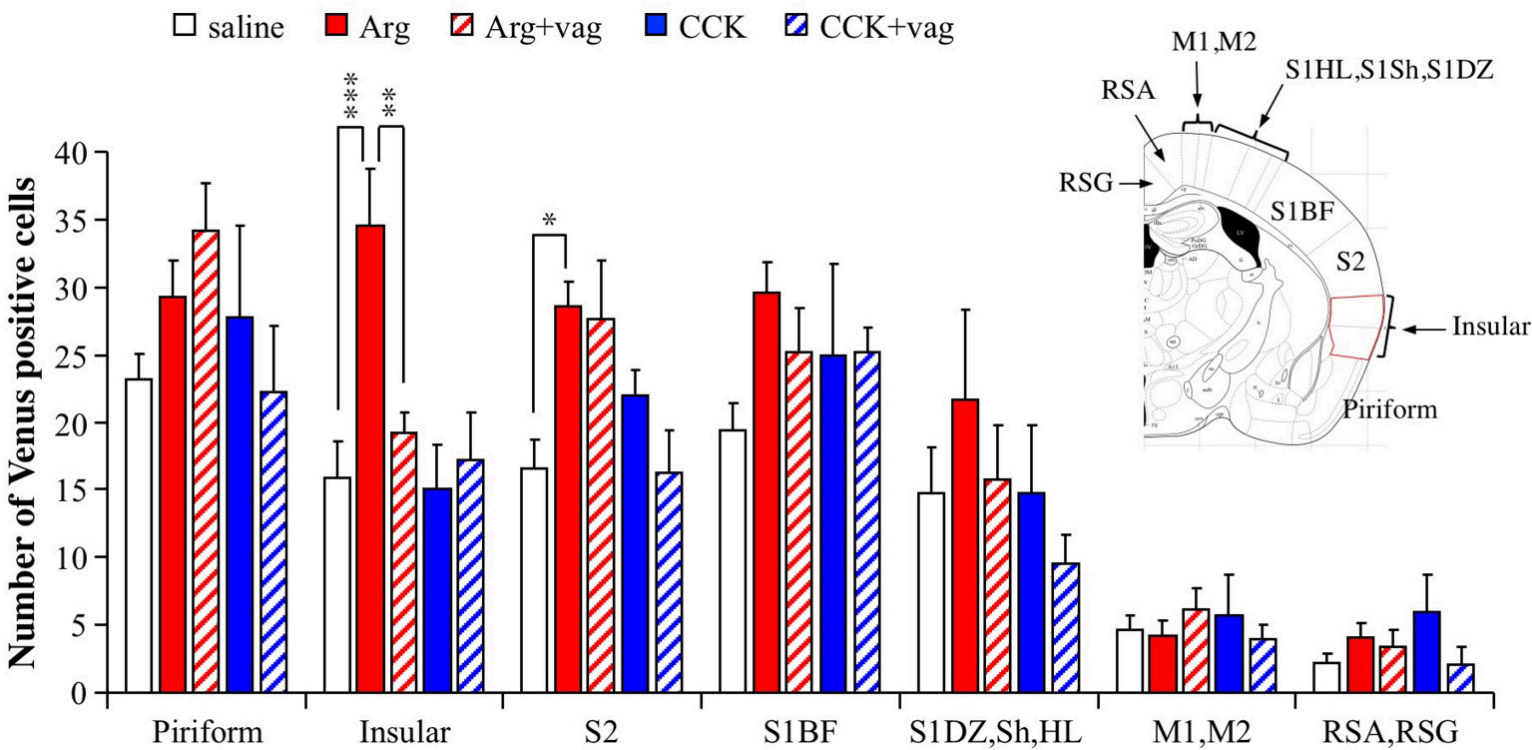
Regional differences in the actions of intraportal administration of Arg (0.61 mmol/kg) and CCK (1.75 nmol/kg) in the cortices of *Arc-dVenus* mice. The numbers of Venus positive cells in the cortices at bregma −1.06 mm were counted in the slices used in the Figure 2 (9 slices from 9 mice for saline, 8 slices from 8 mice for Arg, 7 slices from 7 mice for Arg+vag, 4 slices from 4 mice for CCK, and 4 slices from 4 mice for CCK+vag). Data are shown as mean ± SEM. ^*^P < 0.05, ^**^P < 0.01, ^***^P < 0.001 (*post hoc* Bonferroni test). Note that a vagotomy-dependent increase in the number of Arg-induced Venus positive cells was observed only in the insular cortex. In the S2, the Venus expression induced by Arg was not abolished by vagotomy.

To investigate the regional selectivity of the action of intraportal Arg, we examined the number of Venus positive cells in other cortices observed at the bregma −1.06 mm level, namely the piriform cortex, insular cortex, secondary somatosensory cortex (S2), primary somatosensory cortex (S1) barrel field (S1BF), S1 composed of dysgranular region (DZ), shoulder region (Sh), and hindlimb region (HL), primary and secondary motor cortices (M1 and M2), and region formed by retrosplenial agranular (RSA) and granular (RSG) cortices. The number of mice used were 9 for saline, 8 for Arg, 7 for Arg+vag, 4 for CCK, and 4 for CCK+vag. Except for the insular cortex, there were no regions in which Arg induced an increase in the number of Venus positive cells in a vagotomy-dependent manner [in the insular cortex, *F*_(4, 27)_ = 7.64, *P* = 0.0003 with one-way ANOVA; *P* < 0.001 between saline and Arg and *P* < 0.01 between Arg and Arg+vag with *post hoc* Bonferroni test, Figure 4]. There were significant increases in the number of Venus positive cells after injection of Arg in the S2 [*F*_(4, 27)_ = 3.69, *P* = 0.016 with one-way ANOVA; *P* < 0.05 with *post hoc* Bonferroni test, Figure 4]. However, vagotomy did not significantly reverse the Arg-induced increases in this region (comparison between Arg and Arg+vag, Figure 4). CCK did not induce any clear changes in the number of Venus positive cells in any of the cortical regions examined. These results suggest that there is region-specificity in the CHBV-dependent action of Arg.

### Characterization of Arg action in the insular cortex

**Figure 5:**
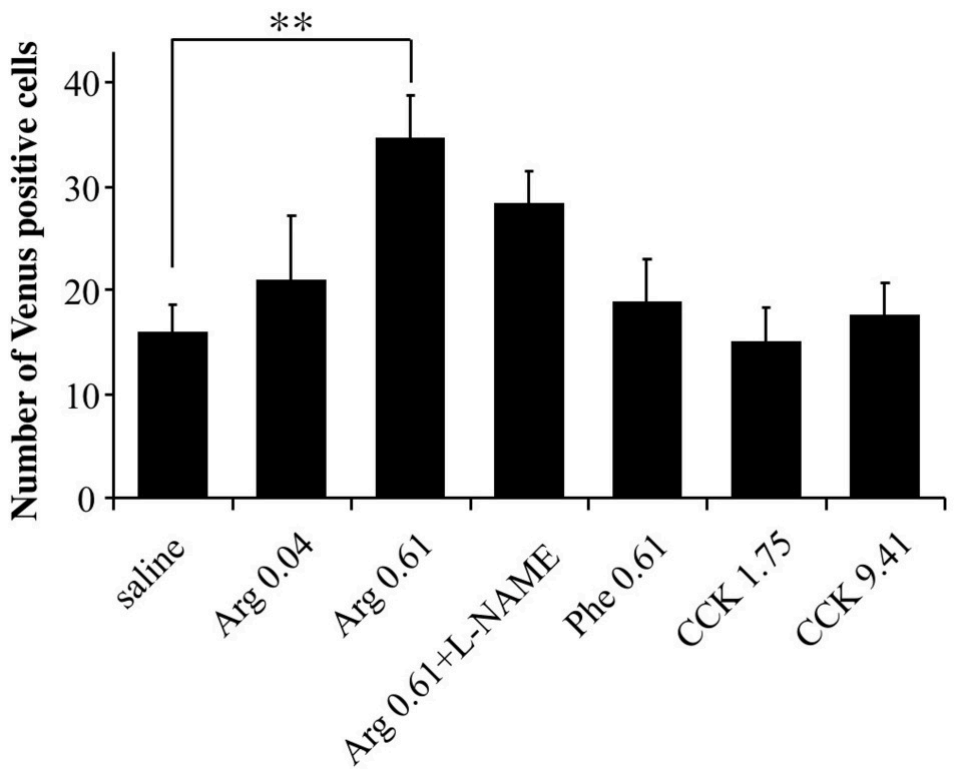
Characterization of the action of Arg in the insular cortex. The dose-dependency in the action of Arg, influence of L-NAME, action of Phe, and action of a high dose (9.41 nmol/kg) of CCK were examined. The numbers of Venus positive cells in the insular cortex were counted in brain slices (bregma −1.06 mm) prepared from *Arc-dVenus* mice intraportally injected with saline or amino acids. The amino acids injected were two doses of Arg (0.04 and 0.61 mmol/kg) and one dose of Phe (0.61 mmol/kg). The data from saline-injected, 0.61 mmol/kg Arg-injected, and 1.75 nmol/kg CCK-injected mice are reproduced from Figure 2. The number of slices used: 9 slices from 9 mice for saline; 6 slices from 6 mice for Arg 0.04 mmol/kg; 8 slices from 8 mice for Arg 0.61 mmol/kg; 3 slices from 3 mice for Arg 0.61 mmol/kg + L-NAME; 5 slices from 5 mice for Phe 0.61 mmol/kg; 4 slices from 4 mice for CCK 1.75 nmol/kg; 4 slices from 4 mice for CCK 9.41 nmol/kg. Data are shown as mean ± SEM. ^**^P < 0.01 (*post hoc* Bonferroni test). Note that the increase in Venus positive cells in the insular cortex was dependent on the dose of Arg, was also observed for co-administration of L-NAME, and was not observed for intraportal Phe and the high dose of CCK.

To characterize the action of intraportal Arg in the insular cortex, we examined (1) the dose-dependency of the action of Arg, (2) the influence of an inhibitor for nitric oxide synthesis from Arg (L-NAME), and (3) the actions of intraportal Phe, which was shown to have no effect on CHBV neurofilament electrophysiological activity (Niijima and Meguid, 1995), in *Arc-dVenus* mouse brain slices at bregma −1.06 mm. The results are summarized in Figure 5, and ANOVA for these data showed significant variation [*F*_(6, 32)_ = 3.26, *P* = 0.013]. The number of mice used were 9 for saline, 6 for Arg 0.04 mmol/kg, 8 for Arg 0.61 mmol/kg, 3 for Arg 0.61 mmol/kg + L-NAME, 5 for Phe 0.61 mmol/kg, 4 for CCK 1.75 nmol/kg, and 4 for CCK 9.41 nmol/kg. The values for saline and Arg 0.61 mmol/kg, and CCK 1.75 nmol/kg were reproduced from Figure 2 for comparison.

First, intraportal Arg at the lower dose (0.04 mmol/kg) did not significantly increase the Venus positive cell number in the insular cortex (Figure 5). Second, L-NAME was dissolved in the Arg 0.61 mmol/kg solution and this mixture was injected into the portal vein (Arg + L-NAME). In these mice, the number of Venus positive cells in the insular cortex (28.3 ± 3.2, Figure 5) did not significantly differ from the number elicited by intraportal injection of Arg 0.61 mmol/kg alone (34.6 ± 4.1). Finally, intraportal Phe 0.61 mmol/kg caused no significant change in the number of Venus positive cells in the insular cortex.

As we mentioned earlier, CCK at 1.75 nmol/kg had no effects on insular Venus expression. However, it remained possible that much higher doses were active. To clarify this issue, we injected a higher dose of CCK (9.41 nmol/kg) into the portal vein, but observed no significant effects on the number of Venus positive cells (Figure 5).

### c-Fos expression in the NTS

**Figure 6:**
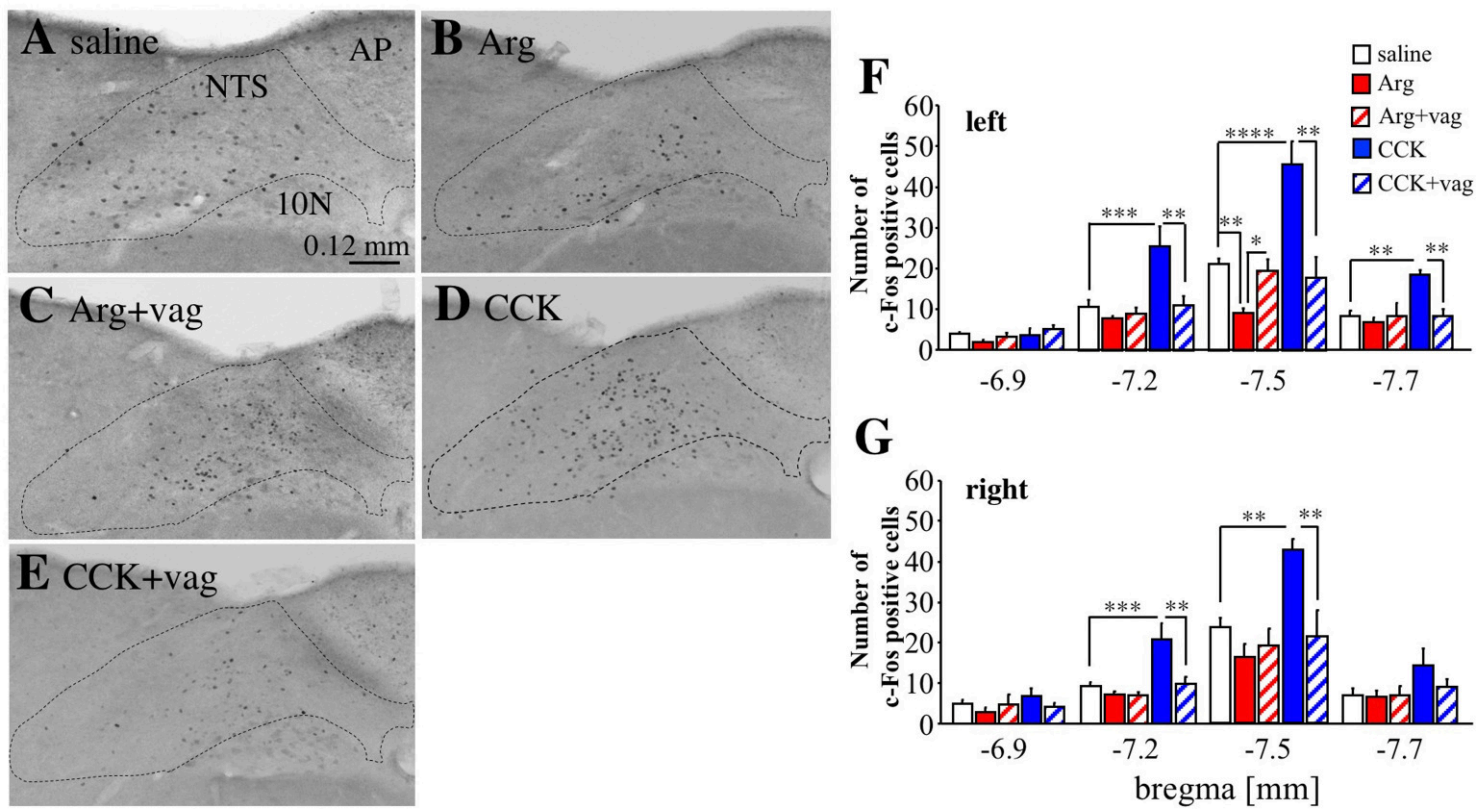
Expression of c-Fos positive cells in the NTS in *Arc-dVenus* mice intraportally injected with CCK or Arg. **(A–E)** Brain slices at bregma −7.5 mm were prepared from intraportally saline-injected **(A)**, Arg (0.61 mmol/kg)-injected **(B)**, Arg (0.61 mmol/kg)-injected and vagotomized **(C)**, CCK (1.75 nmol/kg)-injected **(D)**, and CCK (1.75 nmol/kg)-injected and vagotomized **(E)** *Arc-dVenus* mice used in Figure 2, and immunostained with anti-c-Fos antibodies. These mice were fixed at 180 min after portal vein injection. The dotted lines indicate the NTS. **(F)** The numbers of c-Fos positive cells in the slices at bregma −6.9, −7.2, −7.5, and −7.7 mm were counted within the left medial NTS. **(G)** The numbers of c-Fos positive cells in the slice at bregma −6.9, −7.2, −7.5, and −7.7 mm were counted within the right medial NTS. The numbers of slices used were 6 saline-injected, 5 Arg-injected, 5 Arg+vag, 4 CCK-injected, and 4 CCK+vag for bregma −6.9 mm; 9 saline-injected, 6 Arg-injected, 5 Arg+vag, 4 CCK-injected, and 4 CCK+vag slices for bregma −7.2 mm; 9 saline-injected, 8 Arg-injected, 5 Arg+vag, 4 CCK-injected, and 4 CCK+vag slices for bregma −7.5 mm; 9 saline-injected, 8 Arg-injected, 4 Arg+vag, 4 CCK-injected, and 4 CCK+vag slices for bregma −7.7 mm. Data are shown as mean ± SEM. ^*^P < 0.05, ^**^P < 0.01, ^***^P < 0.001, ^****^P < 0.0001 (*post hoc* Bonferroni test). Note that intraportal Arg induced a significant decrease in the number of c-Fos positive cells in the left medial NTS, while CCK induced a significant increase in the number of c-Fos positive cells in this region.

Next, we analyzed c-Fos expression in the medial NTS, to which the vagal afferents project in the CNS (Figure 6). The expression of c-Fos was analyzed because we could not detect Venus positive cells at all in the NTS. Figures 6A–E show the left NTS at bregma −7.5 mm (the area surrounded by a dotted line). The number of c-Fos positive cells (small black dots) was decreased in the left medial NTS by intraportal Arg (Figure 6A vs. Figure 6B). The number of c-Fos positive cells was not decreased in this Arg-injected vagotomized mouse (Figure 6C) when compared with a saline-injected mouse (Figure 6A). In contrast, intraportal CCK markedly increased this number (Figure 6A vs. Figure 6D), and the increase was not observed in a vagotomized mouse (Figure 6E).

The effects of Arg and CCK in the left medial NTS were examined using several mice, and the results were summarized in Figure 6F (4–9 mice for each group at all four bregma levels, see a Figure 6 legend in detail). In the left medial NTS at bregma −7.5 mm, the mean number of c-Fos positive cells was significantly [*F*_(4, 25)_ = 20.54, *P* < 0.0001 with one-way ANOVA, *P* < 0.01 with *post hoc* Bonferroni's test] decreased by intraportal Arg, which was not observed in vagotomized mice. On the contrary, the mean number of c-Fos positive cells was significantly (*P* < 0.0001) increased by intraportal CCK, which was not observed in vagotomized mice. At bregma −7.2 and −7.7 mm similar effects of intraportal CCK were observed [*F*_(4, 23)_ = 8.35, *P* = 0.0003 with one-way ANOVA, *P* < 0.001 with *post hoc* Bonferroni test for bregma −7.2 mm; *F*_(4, 24)_ = 5.85, *P* = 0.002 with one-way ANOVA and *P* < 0.01 with *post hoc* Bonferroni test for bregma −7.7 mm; Figure 6F] but the effects of Arg could not be observed. At bregma −6.9 mm, neither Arg nor CCK have effects upon the number of c-Fos positive cells. There were no significant changes in the number of c-Fos positive cells in the subnucleus gelatinosus and area postrema at bregma −7.5 mm (data not shown).

In the right NTS (Figure 6G, the number of slices used is the same as the left), on the other hand, the significant decrease in the number of c-Fos positive cells by intraportal Arg was not observed at all in four bregma levels tested. In the case of CCK, the vagotomy-sensitive increase in the number of c-Fos positive cells by intraportal CCK was significant at bregma −7.2 and −7.5 mm, and was not observed at bregma −6.9 and −7.7 mm [*F*_(4, 23)_ = 9.79, *P* < 0.0001 with one-way ANOVA and *P* < 0.01 with *post hoc* Bonferroni test for bregma −7.2 mm; *F*_(4, 25)_ = 6.84, *P* = 0.0007 with one-way ANOVA and *P* < 0.01 with *post hoc* Bonferroni test for bregma −7.5 mm].

These results suggest that intraportal Arg induces the vagotomy-sensitive decrease in the number of c-Fos positive cells in a left NTS specific manner, and that intraportal CCK induces the vagotomy-sensitive increase in the number without outstanding laterality in the NTS.

### c-Fos expression in the hypothalamus

**Figure 7:**
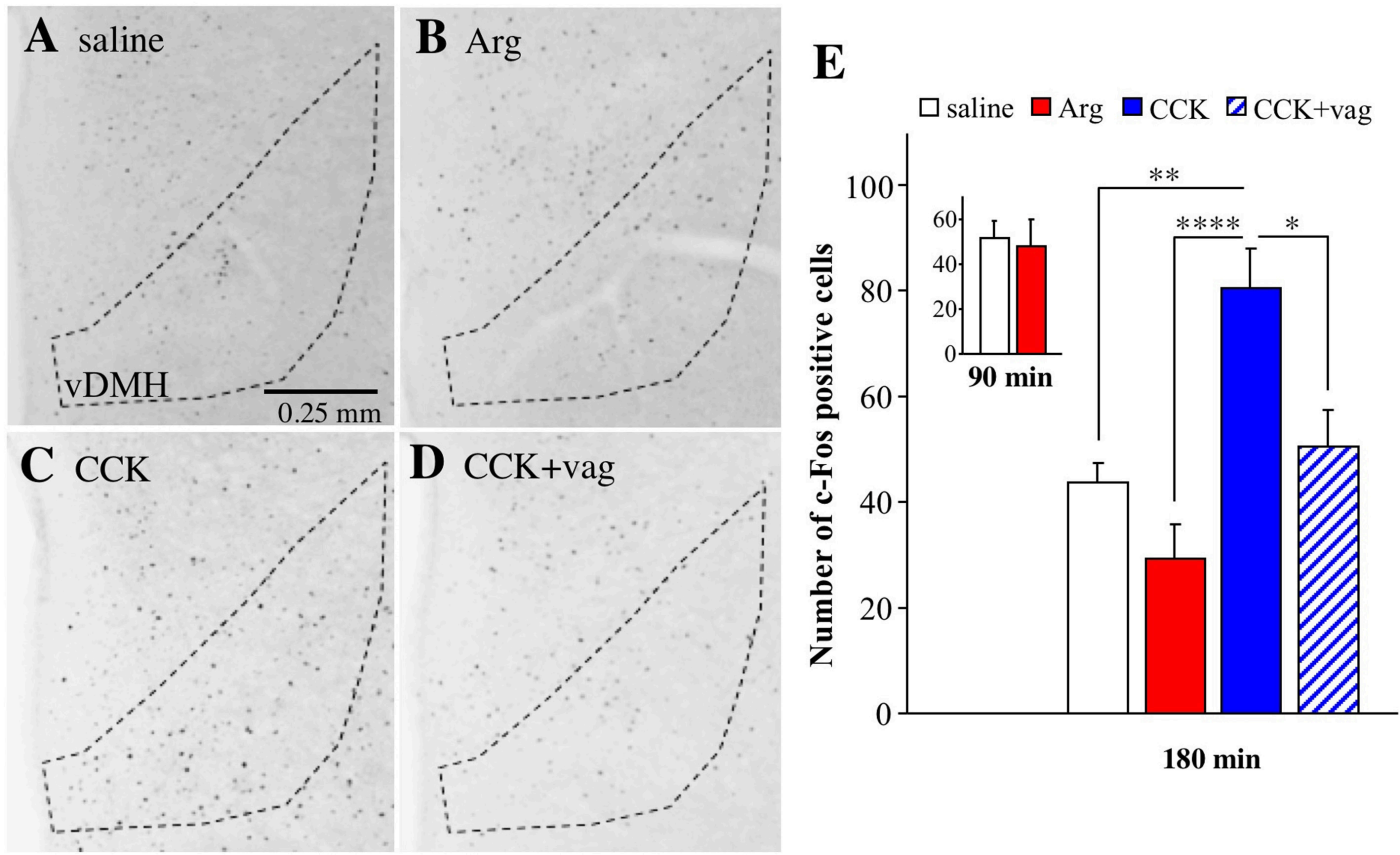
Expression of c-Fos positive cells in the ventral portion of the dorsomedial hypothalamus (vDMH) in *Arc-dVenus* mice intraportally injected with CCK or Arg. **(A–D)** Brain slices at bregma −1.8 mm were prepared from the intraportally saline-injected **(A)**, Arg (0.61 mmol/kg)-injected **(B)**, and CCK (1.75 nmol/kg)-injected **(C)** *Arc-dVenus* mice shown in Figure 2, and immunostained with anti-c-Fos antibodies. **(D)** The brain slices from a vagotomized *Arc-dVenus* mouse intraportally injected with the same dose of CCK were similarly immunostained. The dotted lines indicate the vDMH. These mice were fixed at 180 min after portal vein injection. **(E)** The numbers of c-Fos positive cells in the vDMH in the slices at bregma −1.8 mm were counted in saline-injected (5 slices from 5 mice), Arg-injected (7 slices from 7 mice), CCK-injected (4 slices from 4 mice), and CCK-injected vagotomized (4 slices from 4 mice) mice. (**E-**inset) The data from saline-injected (3 slices from 3 mice) and Arg-injected (5 slices from 5 mice) *Arc-dVenus* mice fixed at 90 min after portal vein injection. Data are shown as mean ± SEM. ^*^P < 0.05, ^**^P < 0.01, ^****^P < 0.0001 (*post hoc* Bonferroni test). Note that intraportal CCK, but not intraportal Arg, induced a vagus-dependent significant increase in the number of c-Fos positive cells in the vDMH.

Finally, we analyzed c-Fos expression in the ventral portion of the dorsomedial hypothalamus at bregma level of −1.8 mm, because peripheral injection of CCK is known to induce c-Fos expression in this region (Kobelt et al., 2006). The expression of c-Fos was analyzed because we could not detect Venus positive cells at all in the hypothalamus. The number of c-Fos positive cells was apparently increased by intraportal CCK (1.75 nmol/kg) in the ventral portion of the dorsomedial hypothalamus, but not by intraportal Arg (comparisons among Figures 7A–C). The effect of CCK was absent in a vagotomized mouse (Figure 7D). The mean number of c-Fos positive cells is summarized in Figure 7E (data from 5 saline-injected mice, 7 Arg-injected mice, 4 CCK-injected mice, and 4 CCK-injected vagotomized mice). One-way ANOVA revealed significant variation in these data [*F*_(3, 16)_ = 11.47, *P* = 0.0003]. The number of c-Fos positive cells was significantly (*P* < 0.01) increased by intraportal CCK, and the action of CCK was significantly (*P* < 0.05) suppressed in vagotomized mice. There was significant difference between Arg-injected and CCK-injected mice (*P* < 0.0001). Meanwhile, there was no significant difference between saline-injected and Arg-injected mice. There was no significant difference between saline- and Arg-injected *Arc-dVenus* mice fixed at 90 min after portal vein injection (3 mice for saline and 5 mice for Arg, Figure 7E-inset). Therefore, it is likely that the absence of the increase in the number of c-Fos positive cells by Arg in the dorsomedial hypothalamus is not due to disappearance of the Arg response at 180 min post-administration.

## Discussion

The main finding of the present study is that intraportal Arg and CCK enhance neuronal activity-dependent IEG expression in distinct brain regions. Specifically, intraportal Arg and CCK enhance the IEG expression in the insular cortex and dorsomedial hypothalamus, respectively, while Arg has no effect on the dorsomedial hypothalamus and CCK has no effect in the insular cortex. The enhancement of IEG expression by Arg in the insular cortex and CCK in the hypothalamus was abolished by CHBV vagotomy. While it has already been shown that the CHBV contains neural fibers projecting to various peripheral organs (Berthoud et al., 1992; Berthoud and Neuhuber, 2000; Horn and Friedman, 2004; Puizillout, 2005), there is little information on the CNS regions activated by this branch. Our results indicate that the CHBV activates different brain regions depending on the type of peripheral stimulation.

One plausible mechanism underlying the enhancement of IEG expression in different brain regions by different compounds is that Arg and CCK activate distinct fibers. These fibers then activate two different neuronal circuits to stimulate the insular cortex or dorsomedial hypothalamus. Consistent with this hypothesis, heterogeneity of the vagus nerve in its sensitivity to chemical substances was observed in single-unit recordings in the rat CHBV (Horn and Friedman, 2004). In these experiments, only three of 28 fibers were stimulated by CCK at sites within the liver or portal vein. Furthermore, jugular vein injection of cisplatin, a cancer chemotherapy agent, electrophysiologically activated vagal afferent fibers in the gastroduodenal branch of the CHBV, but not in the CHBV hepatic branch proper (Horn et al., 2004). Meanwhile, cultured rat nodose ganglion (NG) neurons, where the soma of the vagus nerve is distributed, contain two types of D-glucose-responsive NG neurons. A large proportion of the vagus nerve neurons projecting to the liver are suppressed by D-glucose, while a large proportion of the vagus nerve neurons projecting to the upper gastrointestinal tract are excited by D-glucose (Grabauskas et al., 2010). These findings on the heterogeneity of the vagus nerve neurons are in favor of the possibility that different vagus nerve neurons respond differently to Arg or CCK.

Our data on c-Fos expression in the NTS also indirectly support this notion. Specifically, significant increases in c-Fos immunoreactive signals were observed for CCK, but significant decreases in these signals were detected for Arg only in the left medial NTS. With respect to the laterality, this result of ours is in agreement with a previous result that the CHBV predominantly projects to the left medial NTS in rats (Rogers and Hermann, 1983). However, we could not observe any changes in the number of the c-Fos positive cells in the other regions to which CHBV is shown to project in rats, the left subnucleus gelatinosus and left lateral edge of the area postrema (Rogers and Hermann, 1983). The reason is unclear, but there may be species differences (mouse vs. rat) in the fine projection site of CHBV. Alternatively, CHBV that responds to Arg may project only to the medial NTS. On the other hand, one possible explanation for the increase of c-Fos positive cells by CCK and decrease by Arg is that the vagal fibers activated by CCK application and Arg application innervate different kinds of neurons in the NTS, for example, the former has synaptic contact with NTS excitatory neurons, while the latter has contact with NTS inhibitory neurons. In the NTS, the neuronal circuit in which one GABAergic interneuron projects to multiple glutamatergic neurons is hypothesized (Kawai and Senba, 1996). Such an inhibitory circuit may be participated in the action of Arg to decrease the number of c-Fos positive cells. Further elucidation of the neuronal networks that respond to the CHBV in the NTS is necessary to clarify this issue.

The insular cortex is known as a major cortical region that participates in visceral sensation, suggesting the possibility that it has pivotal roles in interoception of the viscera. The present results provide new evidence that the insular cortex is also one of the targets of the brain neuronal circuits activated by the CHBV. Our results showed that there was no laterality in the action of Arg upon the number of Venus positive cells in the insular cortex. This is in contrast with the clear laterality in the action of Arg in the NTS. It seems that the laterality is lost during ascending projection from the left NTS to the insular.

Our results showed that the number of c-Fos positive cells is consistently larger than the number of Venus positive cells in the insular cortex. This tendency in the ratio of positive cells is also observed in the mouse temporal association cortex (Cho et al., 2016) which localized in the temporal cortex as the insular cortex.

A previous study showed that the dorsomedial hypothalamus receives strong input from the posterior visceral sensory part of the NTS (Ter Horst et al., 1989). The presence of a direct projection from the NTS provides a putative anatomical base for the increase in c-Fos expression induced by CCK in this area, although participation of an indirect projection from the NTS is also possible.

Amino acids produced by the digestion of ingested proteins are absorbed by intestinal epithelial cells and transferred to the liver *via* the hepatic portal vein. Recent studies suggested that amino acids act as signaling molecules in the periphery (Wu, 2013; Pekarova and Lojek, 2015). Since L-NAME, an inhibitor for nitric oxide synthase, had no effects upon the enhancement of Venus expression by intraportal Arg in the insular cortex, it seems that nitric oxide is not participated in the enhancement of Venus expression in the insular cortex. As the insular cortex is known to participate in visceral sensation (Cechetto and Saper, 1987) and has dense reciprocal fiber connections with the amygdala, a center of emotion (Pitkanen, 2000), intraportal Arg at the concentration used in the present study may reflect the formation of implicit emotional feelings after eating. Alternatively, activation of the insular cortex by intraportal Arg may be related to the cardiovascular action of Arg. Stimulation of the insular cortex was shown to induce bradycardia and depressor responses in humans (Oppenheimer et al., 1992). Further investigations are needed to identify the behavioral and physiological outputs of the insular cortex activation by Arg.

In the present study, we focused on the expression of IEGs regulated by Arg or CCK in the insular cortex and dorsomedial hypothalamus, and did not perform a strict survey of the expression of Venus and c-Fos throughout the brain. Therefore, the present study cannot exclude the possibility that there are other brain regions that respond to Arg or CCK.

In summary, we found that administration of Arg and CCK into the mouse hepatic portal vein has effects on the expression of two IEGs, Arc and c-Fos, in the brain. Different brain regions were involved with these effects: the insular cortex for Arg and the dorsomedial hypothalamus for CCK. The present results suggest that the CHBV can activate different brain regions depending on the nature of the peripheral stimulus.

## Author contributions

DY, PK, and MS designed the experiments. DY, PK, SO, and MS performed the experiments. DY, PK, and MS analyzed the data. MS and DY wrote the manuscript. MS and KW developed analytical tools. TY and HK provided reagents. ME and SY provided *Arc-dVenus* mice. All authors discussed the results and implications and commented on the manuscript.

### Conflict of interest statement

The authors declare that the research was conducted in the absence of any commercial or financial relationships that could be construed as a potential conflict of interest.

## Abbreviations

ANOVA: analysis of variance
Arc: activity-regulated cytoskeleton-associated protein
Arg: L-arginine hydrochloride
CCK: cholecystokinin-8
CHBV: common hepatic branch of the vagus
CNS: central nervous system
IEG: immediate early gene
L-NAME: L-*N*^G^-nitroarginine methyl ester
M1: primary motor cortex
M2: secondary motor cortex
NG: nodose ganglion
NT: not tested
NTS: nucleus tractus solitarii
PBS: phosphate-buffered saline
Phe: L-phenylalanine
RSA: retrosplenial agranular cortex
RSG: retrosplenial granular cortex
S1: primary somatosensory cortex
S1BF: S1 barrel field
S1 DZ: S1 dysgranular region
S1 HL: S1 hindlimb region
S1 Sh: S1 shoulder region
S2: secondary somatosensory cortex
SEM: standard error of mean
vDMH: ventral portion of the dorsomedial hypothalamus.
